# Breast Cancer in the Arabian Gulf Countries

**DOI:** 10.3390/cancers15225398

**Published:** 2023-11-14

**Authors:** Humaid O. Al-Shamsi, Nadia Abdelwahed, Amin Abyad, Ibrahim Abu-Gheida, Mehdi Afrit, Tasneem Abu ElFuol, Ryad Alasas, Bilal Lababidi, Prasanta Dash, Mudhasir Ahmad, Norbert W. Dreier, Urfan ul Haq, Thanda Lucy Ann Joshua, Sonia Otsmane, Anwar Al-Nouri, Aydah Al-Awadhi, Syed Hammad Tirmazy, Faisal Alterkait, Shimaa Elsabae, Nyla Khan, Nehad Kazim Albastaki, Yoginee Sonawane, Mohammed Jouda, Frea Perdawood, Faryal Iqbal, Hassan Jaafar

**Affiliations:** 1 Burjeel Medical City, Abu Dhabi P.O. Box 92510, United Arab Emirates; nadia.abdel.wahed.88@gmail.com (N.A.); dr.amin.abyad@gmail.com (A.A.); ibrahim.abugheida@burjeelmedicalcity.com (I.A.-G.); mehdiafrit23@yahoo.fr (M.A.); t.abuelfuol@gmail.com (T.A.E.); ryadalasas@gmail.com (R.A.); bilabak@gmail.com (B.L.); dr.prasantadash@gmail.com (P.D.); drmudhasir@hotmail.com (M.A.); norbert.dreier@burjeel.com (N.W.D.); drirfan1@yahoo.com (U.u.H.); thandajoshua@gmail.com (T.L.A.J.); soniayk@hotmail.com (S.O.); shoshoelsaba@yahoo.com (S.E.); nyla.a.khan@gmail.com (N.K.); drnehad.kazim@gmail.com (N.K.A.); dryoginees28@gmail.com (Y.S.); mohammad.judah74@gmail.com (M.J.); frea73@yahoo.com (F.P.); faryal.iqbal@burjeelmedicalcity.com (F.I.); hassan.jaafar@burjeelmedicalcity.com (H.J.); 2 Emirates Oncology Society, Dubai P.O. Box 6600, United Arab Emirates; ayawadhi@seha.ae; 3 Department of Clinical Sciences, College of Medicine, Gulf Medical University, Ajman P.O. Box 4184, United Arab Emirates; 4 Department of Clinical Sciences, College of Medicine, University of Sharjah, Sharjah P.O. Box 27272, United Arab Emirates; 5 Kuwait Cancer Control Center, Kuwait City, Kuwait; alnourioncology@yahoo.com (A.A.-N.); falterkait@moh.gov.kw (F.A.); 6 Dubai Hospital, Dubai P.O. Box 7272, United Arab Emirates; syedtirmazy@gmail.com

**Keywords:** breast cancer, malignancy, Gulf Cooperation Council (GCC), survival, HER2, triple negative

## Abstract

**Simple Summary:**

Breast cancer is the most frequently diagnosed cancer in all six countries of the Gulf Cooperation Council (GCC). In our research, our focus was on understanding how often breast cancer occurs and how this pattern has evolved within the GCC. The authors’ objective is to shed light on the challenges related to the changing landscape of breast cancer and to raise awareness among healthcare leaders and individuals working in these affected regions about areas that need improvement. We identified that in the past, there were difficulties in accurately tracking breast cancer cases, but there has been recent progress with more detailed reporting in national cancer registries. Nevertheless, there remains a shortage of information, particularly regarding the spread of breast cancer, treatment methods, and patient outcomes. The limited available data are mostly outdated and may not accurately reflect the current status of breast cancer in the GCC. This is because medical approaches to breast cancer treatment and patient outcomes are continually evolving with the introduction of new therapies.

**Abstract:**

Breast cancer stands as the prevailing malignancy across all six Gulf Cooperation Council (GCC) nations. In this literature review, we highlighted the incidence and trend of breast cancer in the GCC. Most of the studies reported a consistent increase in breast cancer incidence over the past decades, which was particularly attributed to the adoption of a Westernized lifestyle in the region and the implications of emerging risk factors and other environmental and societal factors, the increase in screening uptake, as well as the improvement in data collection and reporting in the GCC. The data on breast cancer risk factors in the GCC were limited. In this geographic region, breast cancer frequently manifests with distinctive characteristics, including an early onset, typically occurring before the age of 50; an advanced stage at presentation; and a higher pathological grade. Additionally, it often exhibits more aggressive features such as human epidermal growth factor receptor 2 (HER2) positivity or the presence of triple-negative (TN) attributes, particularly among younger patients. Despite the growing body of literature on breast cancer in the GCC, data pertaining to survival rates are, regrettably, meager. Reports on breast cancer survival rates emanating from the GCC region are largely confined to Saudi Arabia and the United Arab Emirates (UAE). In the UAE, predictive modeling reveals 2-year and 5-year survival rates of 97% and 89%, respectively, for the same period under scrutiny. These rates, when compared to Western counterparts such as Australia (89.5%) and Canada (88.2%), fall within the expected range. Conversely, Saudi Arabia reports a notably lower 5-year survival rate, standing at 72%. This disparity in survival rates underscores the need for further research directed toward elucidating risk factors and barriers that hinder early detection and screening. Additionally, there is a pressing need for expanded data reporting on survival outcomes within the GCC. In sum, a more comprehensive and nuanced understanding of breast cancer dynamics in this region is imperative to inform effective strategies for prevention, early detection, and improved patient outcomes.

## 1. Introduction

Recently, breast cancer (BC) has surpassed lung cancer to become the most diagnosed cancer in the world, with approximately 2.3 million new cases diagnosed in 2020, while still globally contributing to the top five leading causes of cancer mortality [1]. It is commonly known that BC incidence rates in Gulf Cooperation Council (GCC) countries are lower than in Westernized countries; nevertheless, it is postulated that the anticipated demographic shift from a younger to an older population, increasing life expectancy, and the unique and evolving combination of lifestyle factors in the GCC would lead to a substantial increase in cancer incidence and mortality, including BC, over the next two decades [2].

Breast cancer is the most common cancer among women. In light of the anticipated increase, proper planning and decision making require reliable, up-to-date, and accurate data to ensure effective cancer control and the provision of optimal care for patients with BC. We, therefore, aimed to review the available literature from the past decade on BC in the GCC to compile, summarize, and broadly evaluate the available evidence with the aim of highlighting existing gaps and needs in BC research in the region and accordingly inform and prioritize future regional and country-level research strategies.

## 2. Literature Review

We performed a literature search to review and collect research and/or reports on BC epidemiology, diagnosis, and management published between 2011 and 2022 from the GCC countries, with a focus on the UAE, Qatar, Kuwait, Bahrain, Oman, and the Kingdom of Saudi Arabia. We reviewed PubMed/Medline and Google Scholar for peer-reviewed articles, searched the ASCO and ESMO congresses for relevant abstracts presented during the past 5 years, and included the most recent GLOBOCAN and national cancer registry reports for each of the six countries.

## 3. Epidemiology of BC in the GCC

We extracted BC epidemiological data from three sources: (1) The GLOBOCAN database [3], which estimates data based on projection methods and modeling and is highly dependent on the quality of data in the corresponding or neighboring countries. (2) The national cancer registries; these were not always consistent in their reporting and publishing of yearly and updated data and, similar to the GLOBOCAN data, are highly dependent on the quality of surveillance data in the countries and the coverage and completeness of the data. The most recent reports published from these registries were from 2019 for the UAE [4], 2018 for Qatar [5], 2018 for Bahrain [6], 2019 for Oman [7], 2017 for Kuwait [8], and 2015 for Saudi Arabia [9]. Available data from these reports and the GLOBOCAN database are presented in Table 1. It was evident from these reports that BC was the leading cancer in all six countries; nevertheless, country comparisons of incidence rates and other figures are difficult due to differences in the year of reporting, the reported measures, and the considered populations (nationals vs. non-nationals). (3) Finally, epidemiological studies are another source of epidemiological data, but looking back at the past 10 years, such studies have been very scarce. Most of the studies that we reviewed were small-scale, single-center studies or systematic reviews looking at GCC or Arab countries collectively, reporting on historical data, and which are unlikely to be reflective of the actual BC status in the individual countries. However, these studies can usually hint at trends in BC incidence and other measures, which are summarized herein.

### 3.1. Incidence

Most of the studies reported a consistent increase in BC incidence over the past decades, which was particularly attributed to the adoption of a Westernized lifestyle in the region and the implications of emerging risk factors and other environmental and societal factors, as well as to the increase in screening uptake [10]. Both citizens and non-citizens of the GCC region experienced breast cancer earlier than women in comparable high-income nations [11].

Data from the Kuwait Cancer Registry showed that the annual number of new cases remarkably increased from 212 cases in 2012 to 608 cases in 2017 [8,12]. The age-standardized rate (ASR) was 61.0 and 41.3 cases per 100,000 populations for Kuwaiti and non-Kuwaiti females, respectively. Breast cancer among males represents <1.0% of breast cancer cases [8]. As for Kuwaiti females, BC had the highest incidence; it increased by three-fold over the last 44 years (18.5 to 63.5 cases per 100,000 people per year). The trend of the ASR of Kuwaiti female breast cancer cases over the time period of 2013–2017 was 64.6 per 100,000, where the crude rate was 47.85 per 100,000, while the trend of non-Kuwaiti female breast cancer cases over the time period was 45.2 per 100,000, and the crude rate was 29.06 per 100,000 [8].

A systematic review looking at cancer trends in the UAE confirmed that BC ranked first among female cancers between 1998 and 2001, with an ASR ranging between 17.1 and 19.2 per 100,000 [13]. As of 2019, there were 883 cases of BC in the UAE, accounting for 20.2% of all malignant cases, according to more recent data from the National Cancer Registry of the UAE [4]. Data from the Arab region (including, among others, Bahrain, Kuwait, Oman, Qatar, and the UAE) also described a gradual rise in BC cases between 1990 and 2016, similar to the global trend, and reported a BC incidence of 28 per 100,000 in 2016 [14]; nevertheless, the authors noted that, although high, the incidence was still lower than the global incidence of 46 per 100,000 and remarkably lower than the incidence in Western Europe (148 per 100,000). Also, the burden in terms of disability-adjusted life years was lower in Arab countries compared with Western countries [14]. The most recent available data from a systematic review and meta-analysis of 80 studies from the Eastern Mediterranean Region (including Qatar, Kuwait, Oman, Bahrain, and the UAE) reported a pooled ASR of 37.1 per 100,000 person-years (95% confidence interval (CI), 34.5, 39.8) during 2011–2019 and an increasing trend between 2005 and 2019. However, the trend varied between countries and regions based on the quality of the data [12]. Data from the Qatar National Cancer Registry in 2018 show that Qatar has an ASR of 87.07 per 100,000 of the female population at risk, whereas the Saudi Cancer Registry from 2015 shows an ASR of 24.3 per 100,000 (among Saudi females) and 29.8 per 100,000 (among non-Saudi females) [5,9]. The Oman Cancer Registry shows the highest age-specific incident rate for BC among the Omani female population of 112.2 at the age of 50 [7].

### 3.2. Risk Factors

Research about BC risk factors in the six countries was limited. Our search identified one study from Qatar describing the use of BC risk assessment to better guide the clinician’s decision about screening and clinical management. This study used the Gail model to evaluate the BC risk among 1338 women aged 35 years and older selected from 12 primary healthcare centers in Qatar between July 2012 and June 2014. This study reported mean 5-year and lifetime BC risks of 1.12 ± 0.52 and 10.57 ± 3.1, respectively, among the women of Qatar. A linear regression analysis identified significant predictors of the 5-year and lifetime risks, such as age, age at menarche, age at first birth, family history, and age at menopause. Overall, the Gail model was found to be an appropriate risk assessment tool in the Gulf. Nevertheless, one of its limitations, as reported by the authors, is that it does not take into consideration BC among second-degree relatives as a risk factor, and it may incorrectly estimate the risk of BC as reported in previous studies given that risk factors differ between ethnicities [15]. A systematic review looking at the association between obesity, physical inactivity, and BC incidence in the GCC did not report any association between BC and neither obesity nor physical inactivity in the five countries of interest between 1999 and 2019 [16]. One study looked at the association between serum 25-hydroxyvitamin D [25(OH)D] levels and BC risk and found that women with serum 25(OH)D levels lower than 20 ng/mL were at higher risk of BC (odds ratio, 4.63; 95% CI, 2.61–8.23) after adjusting for age, body mass index, and menopause status [17].

### 3.3. Financial Burden

In terms of financial burden, a review looking at the economic costs associated with non-communicable diseases (NCDs), including BC in the GCC, concluded that only 0.5% of the annual total direct medical costs of selected NCDs, which were estimated at USD 16.7 billion, were due to BC, implicating that BC was less common compared with other NCDs such as type 2 diabetes mellitus and coronary heart disease [18].

## 4. BC Presentation in the Region

Data from the national cancer registries provide limited insights around the presentation of BC in the region, given that they are very heterogeneous in terms of collected data, population coverage (nationals vs. non-nationals), and completeness; are not up-to-date; and therefore do not provide an accurate description of the clinicopathologic features of BC in the region that may be evolving over time. Some clinicopathologic features retrieved from the corresponding national registries are presented in Table 2.

There were quite a few studies (single centers, reporting on findings from the national registries, or even systematic reviews) describing clinicopathologic features of BC in the five respective countries. Overall, it was consistently reported across these studies that in the region, BC was characterized by an early onset (generally before 50), an advanced stage, and a higher pathologic grade, as well as more aggressive features such as human epidermal growth factor receptor 2 (HER2) positivity or triple-negative (TN) features, especially in young patients.

### 4.1. Age at Diagnosis

In Oman, a study reported that more than half of the 14,109 cancer cases (53.5%) diagnosed between 1996 and 2010 were diagnosed below 50 years of age, and the highest age-specific incidence rates were between 50 and 54 years (57.1 per 100,000) and 45 and 49 years (46.7 per 100,000) [19]. Through Oman Cancer registry sources, the highest frequency of incident cases among Omani female nationals was at the age of 35 (*n* = 56) [7]. In the UAE, the average age at diagnosis among the 988 patients diagnosed with BC at Tawam Hospital between January 2008 and December 2012 was 48 years [20]. Another, more recent study from Sharjah Breast Care Centre reported a mean age at diagnosis of 51 ± 12 years in 94 patients diagnosed with BC between March 2016 and July 2018 [17]. Data from Bahrain from the periods 2000–2010 and 2017–2019 reported a mean age at diagnosis of 50.9 and 51.8 in 1005 [21] and 216 BC patients, respectively [22]. The median age at diagnosis of female breast cancer among the Saudi female population was 50 years (ranged between 14 and 108 years), and the number of BC incident cases peaked in the age group of 45–49 (*n* = 356) [9].

The GCCR recorded that 25.5% of all BC cases occurred in women less than 40 years old in the GCC [23], and a review reported an overall mean age at BC diagnosis of 50.6 years in the GCC countries compared with 60 years in Western countries [10]. Another review from the Arab countries mentioned that the median age at diagnosis in the Arab population was about 48 years and that about two thirds of women with BC were younger than 50 years [24]. These review papers also highlight the early onset of BC among Arab countries and the GCC in comparison with Western populations, where the highest age-specific incidence rates were in the higher age groups, and explain that this might be primarily due to a younger population structure in the region as well as to differences in environmental factors, genetic makeup, screening practices, and disease management [10,24]. One study, however, did not find any significant difference in BC incidence during 1990–2016 between younger women in Arab countries (30–59) and their global counterparts, while rates among older women (60 and above) were significantly lower [14]. 

### 4.2. Prognosis and Survival

To our knowledge, a single-institutional retrospective analysis analyzing 988 patients with a follow-up of 35 months was the only study examining BC survival in the UAE. The predicted 2-year and 5-year survival rates for the same period were 97% and 89%, respectively, and were within the normal ranges when compared to countries in the Western world such as Australia (89.5%) and Canada (88.2%). Comparing Kuwait (75.2%) and Qatar (71.95%), two countries in the same region, the 5-year survival rate is also high [20,25,26]. According to the 2017 annual report of the Kuwait Cancer Registry, the overall 5-year survival rate of Kuwaiti BC patients was 90.5% [8]. According to Kuwait’s NCR status at the last follow-ups, 95% of Kuwaitis were live and 5% were dead; meanwhile, 98% of Kuwaitis were live and 2% were dead [8]. In the Qatar National Cancer Registry (QNCR), 894 incidences of female breast cancer were identified among the Qatari population; out of them, 699 (78%) are still alive [5].

Breast cancer represents the commonest cancer in Saudi females and in the Saudi population in general. Despite a 5-year observed survival of 72%, this represents a slightly lower value than western countries, though most of them report relative 5-year survival [25,27,28]. Reasons behind a lower survival are likely related to a relatively high percentage of presentation in advanced stage (12.5%) and a low rate of screening [28,29]. Screening for breast cancer has developed in several phases yet has not materialized into a national screening program [28,30,31,32,33]. Several opportunistic screening campaigns took place in several cities of Saudi Arabia mostly by non-governmental organizations [28,33].

### 4.3. Stage and Grade

The GCCR reports that up to 58% of the patients present at a late stage with regional or distant metastases [23]. A review of data from the Kuwait Cancer Registry showed that there was an overall decreasing trend in localized and regional stages between 2000 and 2004, 2005 and 2009, and 2010 and 2013, and an increasing trend in distant and unknown stages [34]. About 39.3% of all breast cancers presented with regional extension, 13.9% presented with distant metastasis, and an unknown extent of disease was observed in about 22.7%. Stage II was the most frequent presentation at diagnosis: 28.0% [8]. A report from the Kuwait Cancer Registry shows the following stage distribution for breast cancer cases diagnosed in 2017 among Kuwaiti nationals, stage I: 17.0%, II: 29.0%, III: 20.0%, IV: 12.0%, unknown: 21%, and Tis: 1%, whereas that among non-Kuwaiti nationals was stage I: 10%, II: 27%, III: 28%, IV: 16%, Unknown: 16%, and Tis: 3% [8]. Although the UAE Cancer Registry does not have complete data for the stage at presentation, it is evident that there is a relative decline in stage IV disease, with the percentage of localized disease increasing from 10% in 2011 to 25% in 2017 [4]. This may reflect improvements in mammography screening and uptake because of public awareness [35]. A report from the Oman Cancer Registry shows an increasing trend for stage I: 13% and stage II: 27% from 2018 to 2019, where 2019 data show stage I: 27% and stage II: 34%, while Stage III shows a drastic decline from 30% in 2018 to 17% in 2019 [7].

It is discussed that the stage at diagnosis is largely driven by the effectiveness of available screening and public awareness programs in the country and that the high number of cancer cases with an “unknown” stage might be associated with the unique biology of BC in the Arab region [10].

### 4.4. Histology

In terms of histological subtypes, all the reviewed studies confirmed the data from the national registries’ reports [4,5,6,7] with infiltrating ductal carcinoma being the most common BC histology [10,17,21,24,36,37]. Among the respective recent national cancer registries of Qatar, Bahrain, Oman, and Saudi Arabia, Oman has the highest rate of infiltrating duct carcinoma at 90.3% [5,6,7,9].

### 4.5. Molecular Subtypes

In Oman, the luminal A subtype was the most common among 542 cases of BC diagnosed between 2006 and 2010 [19]. Similarly, luminal A was the most common in Bahrain (60.2% of 216 patients), followed by luminal B (19%), TN (13.4%), and HER2+ (7.4%) [36]. In the UAE, a short letter to the editor reported an overall incidence of estrogen-receptor-positive (ER+), progesterone-receptor-positive (PR+), HER2+, and TN tumors of 59.3%, 51.0%, 39.1%, and 20.8%, respectively, among the 192 patients diagnosed with BC between April 2008 and May 2009 [38]. More recently, a study reported positive hormone receptor (HR) status in 62 out of 94 patients with BC (66.0%) between 2016 and 2018 [17]. Additionally, data from MD Anderson Cancer Center on 78 Arab women diagnosed between 2010 and 2018 revealed an ER positivity of 69.2% and a PR positivity of 65.4%. HER2+ status was reported in 19.2% of the patients, while 26.9% had TN disease [39]. Data from the KCCC reported TN disease in 363 patients out of 2980 (12.2%) between 1999 and 2009 [40,41]. One paper looked at the changes in the biological features between primary and recurrent tumors in 70 patients who presented between 2009 and 2012 in Kuwait, and it reported a decrease in ER and PR positivity from 61.4% to 58.6% and 61.4% to 44.3%, respectively, with an increase in HER2 positivity in 5.7% of cases [42].

### 4.6. Association of Clinicopathologic Features with Selected Factors

Several studies looked at the association between clinicopathologic characteristics and selected factors. The most consistent finding across these studies was the observation that younger patients were more likely to have HER2+, TN, or basal-like cancers as compared with older patients [17,22,38,43]. Additionally, younger patients were more likely to have tumors of a higher stage (*p* = 0.012) and grade (*p* = 0.031) and were more likely to have lymph node metastases (88.6% versus 56.1%) (*p* = 0.0004) and distant metastases (26.7% versus 6.8%) (*p* = 0.005), leading to a worse prognosis [22]. Conversely, a study from Bahrain (*n* = 1005) did not find any association between age at diagnosis and tumor grade, metastasis, or stage of cancer [21]. One study from Oman observed that high-grade tumors were most common in the basal-like subtype (41.0%) and lowest in the luminal A subtype (19.0%), while a higher stage at presentation (stages III and IV) was more common in HER2+ tumors (59.0%) [43]. Looking specifically at the TN subtype, a study from Bahrain (*n* = 216) found that this subtype was associated with higher-grade tumors compared with other subtypes (*p* = 0.001) [36], which was confirmed by an interim analysis from the TRIPOLI study, which demonstrated that compared to patients > 40 years, patients ≤ 40 years were more likely to have grade 3 tumors (62.3% versus 53.5%; *p* = 0.116) and more likely to have stage III/IV tumors (41.4% versus 32.7%; *p* = 0.038) [37]. Finally, one study from Qatar explored the co-prevalence of high-risk human papillomavirus (HPV) and Epstein–Barr virus (EBV) in 74 BC tissues and found that the presence of HPV was associated with TNBC (*p* = 0.008), while the co-presence of HPV and EBV was significantly associated with luminal A subtype (*p* = 0.02), tumor grade (*p* = 0.04), and tumor stage (*p* = 0.04) [44].

## 5. Genetic and Molecular Profiling

### 5.1. BRCA1/2 and Other Genes

The involvement of BRCA mutations in familial and sporadic BC has been fairly explored in the GCC, but findings have been inconclusive across studies, and other genes have been suspected to be implicated in the onset or progression of BC, of which BRIP1 was the most reported.

A study looked at the frequency of BRCA1/2 mutations in high-risk affected and unaffected patients referred to the hereditary and high-risk clinic in Qatar between March 2013 and December 2016 based on either a personal or family history of breast and/or ovarian cancers. Of the 167 subjects who underwent genetic testing, 38.0% had BRCA mutations, 41 of whom had BC [45]. A study from Bahrain identified two deleterious mutations, BRCA1 c.4850C>A and BRCA2 c.67 + 2T>C, in two patients who appeared to have a strong family history of BC, with an overall prevalence rate of 8.0% in 25 unrelated females diagnosed with familial BC [46]. In the UAE, among 309 patients with BC undergoing genetic testing from 2016 to 2018, 19 positive susceptibility genes were identified in 130 cases who tested positive for mutations. In 34.6% of the cases, pathogenic and likely pathogenic variants were identified, with BRCA2 being the most commonly identified mutated gene in 29 cases, followed by BRCA1 in 25 cases. A positive family history of BRCA1/2 mutations was found in 29 cases out of 66 (53.7%) with a family history of BC [47]. 

In contrast, other studies only found a minimal to no role for BRCA mutations in BC, with the involvement of a variety of other genes. A study from Oman looking at 50 BC patients and 30 healthy individuals and/or carriers of benign tumors undergoing biopsy and BC surgery between January 2012 and April 2014 found that the majority of patients (84.0%) did not have a family history of BC and did not exhibit any BRCA1/2 mutations, indicating that such mutations are unlikely to play a key role in sporadic BCs [48]. An earlier study did not detect any significant mutational rates in BRCA1/2 either [49], which led to the investigation of other genes linked to BRCA1/2 that could be involved in the onset of BC, such as BRIP1. BRIP1 was found to potentially interact with BRCA1/2 as an oncogene, albeit previously demonstrated as a tumor suppressor gene. It was expressed five times more in breast tumors as compared to normal tissue. Further analyses showed that BRIP1 overexpression was associated with poor overall survival (OS) and with the luminal A and B subtypes [48]. A cross-sectional study that looked at 78 Arab women diagnosed with BC at MD Anderson Cancer Center between January 2010 and December 2018 and who underwent standardized hotspot mutation testing using 46- or 50-gene multiplexes, identified five somatic mutations that were most expressed in these patients: TP53 (23.1%) and PIK3CA (15.4%), followed by PTEN, APC, and KIT (7.7% each). The germline BRCA1 mutation was reported in 8/27 (29.6%) patients while no germline BRCA2 mutations were identified [39]. One study from Oman, using microarray gene expression profiling to analyze gene expression in luminal A, luminal B, and TN breast tumors compared with normal or benign breast tissue, identified more than 1000 differentially expressed genes potentially associated with signaling pathways playing a key role in the malignant transformation of benign tumors [50]. Further, a first-of-its-kind study of the RNA-Seq transcriptome analysis of BC from the GCC region using next-generation sequencing and the ingenuity pathway analysis platform to understand signaling aberrations in BC biology identified 1108 upregulated and 518 downregulated transcripts when the transcriptome of BC was compared to adjacent normal tissue from six BC patients. Additionally, BC tissue exhibited a marked enrichment in genes, promoting cellular proliferation, migration, survival, and DNA replication and repair [51]. Other studies looked at the role of some other genes, such as IGF1 cytosine adenine (CA) [52] and p53 [53], in BC risk, while a sequencing study from Qatar analyzed the role of ER 1 gene mutations on drugs binding affinity to ER [54].

### 5.2. Value of Genetic Profiling

We identified two studies from the UAE and Kuwait looking at the value of Oncotype DX testing in clinical practice and treatment decisions. One study looked particularly at the value of Oncotype DX testing on adjuvant treatment decisions based on a retrospective analysis of data for 50 female node-negative ER+ early BC patients who underwent Oncotype DX between October 2009 and June 2012 in the UAE. The test was successful in 47 patients, and the proportion of patients with low, intermediate, and high recurrence scores was 53.2%, 40.4%, and 6.4%, respectively. The risk assessment based on the St. Gallen criteria and Oncotype DX testing were concordant in approximately half of the patients. Treatment decisions were changed (pre-testing vs. post-testing) in 28% of patients, mostly from chemoendocrine therapy to endocrine therapy alone, with a statistically significant change in the low recurrence group (from 56.0 to 8.0%; *p* = 0.0005) [55]. The second study, from Kuwait, showed that of 100 patients, the Oncotype DX assay resulted in a change in treatment recommendations in 37.0% of patients, of whom 29 were recommended endocrine therapy instead of chemoendocrine therapy. In addition, this study demonstrated a strong association between recurrence score and clinicopathologic features such as tumor grade, Ki67 index, and luminal type. This study concluded that, based on these observations, these features can guide the use of Oncotype DX in specific groups of patients [56].

### 5.3. Other Biomarkers

We found several studies from the GCC looking at the significance of specific markers in the presentation and prognosis of BC. A study from Oman found a strong association between metastasis and younger age (women ≤ 40 years), lymphovascular invasion, and epithelial–mesenchymal transition (EMT) expression, indicating that EMT could possibly predict a higher metastatic potential in tumors and suggesting EMT expression as a surrogate marker for predicting metastasis [57]. In addition, EMT was found to be associated with Ki67 PI and basal-like tumors [57]. Further, the expression of nucleostemin was found to be higher in less differentiated, more advanced stage, larger, and lymph-node-positive tumors, as well as in more aggressive molecular subtypes (HER2+ and TN) when evaluated in 51 patient archival specimens from the UAE, although none of these associations reached statistical significance [58]. A study from Oman observed a statistically significant relationship between high elastosis and ER positivity (*p* = 0.015) and HER2-status (*p* = 0.045) in 80 female patients who were not treated with neoadjuvant therapy from 2009 to 2019, suggesting that elastosis may be used as a surrogate marker for ER positivity and HER2 negativity in BC [59]. A study from the UAE found that A20 expression evaluated through immunohistochemistry (IHC) was associated with early grade 1 BC (*p* < 0.001) in all molecular subtypes, suggesting its use as a biomarker for early cancer. A20 overexpression was also associated with a lower OS rate in patients treated with endocrine therapy [60]. Another study found that insulin-like growth factor 1 receptor (IGF1R) membranous and mixed (membranous and cytoplasmic) expression in BC cells was evident in HR + HER2− cases in contrast with HR−HER2+ cases, which showed cytoplasmic or diminished IGF1R expression, suggesting that luminal subtypes may benefit from targeted IGFR therapy [61]. Trefoil factor 3 (TFF3) expression was found to be associated with residual breast carcinoma following neoadjuvant chemotherapy in 133 cases from the UAE, suggesting that its expression is associated with increased resistance to chemotherapy. Moreover, there was a significant co-expression of TFF3 with antiapoptotic proteins AKT1 (*p* = 0.0365), BCl2 (*p* = 0.0152), and NF Kappa-B (*p* = 0.0243) in breast carcinoma cases with residual carcinoma following neoadjuvant therapy, which reinforces the role of TFF3 in chemoresistance [62]. Vitamin D receptor (VDR) was also assessed using IHC in 120 Kuwaiti female BC fixed tissues and was found altered in BC, with its absence being associated with high-grade differentiated tumors (*p* = 0.01) and its cytoplasmic expression being associated with lymph-node-positive tumors (*p* = 0.03) [63]. A study from Oman demonstrated that the expression patterns of Akt1, E2F1, and their phosphorylated forms in BC tissues correlated with clinicopathological characteristics, cancer progression, overall survival (OS), and response to chemotherapy [64].

A study from the UAE found that the immune profile of the tumor microenvironment (TME) of BC patients is not reflected in the circulation and that there is no association between levels of circulating myeloid cells and patients’ TNM stage or histological grade, highlighting the role of myeloid cells specifically in the TME of BC patients but not in the peripheral blood and suggesting some potential therapeutic modalities to target the expanded immunosuppressive populations in the TME [65].

## 6. Management and Outcomes

### 6.1. Management Trends

Data on BC management trends in the region were very scarce and far from representative of current practice. In the 2018 QNCR, 319 (89%) of the total cases were reported with treatment information. Among them, the top five treatment modalities were Chemotherapy/Surgery (23.20%); Surgery (19.12%); Chemotherapy/Radiation Therapy/Surgery (15.67%); Chemotherapy/Hormonal Therapy/Radiation Therapy/Surgery (12.23%); and Hormonal Therapy/Radiation Therapy/Surgery (8.78%) [5], while data from the KCCC reported the treatment type for 359 patients with TNBC between 1999 and 2009 [40]. Recently, KCCC reported the type of treatment among Kuwaiti and non-Kuwaiti for the year 2017, where 82.3% of Kuwaiti nationals had surgery; 60.8% had chemotherapy; 65.8% had radiotherapy; and 74.0% had hormonal-based treatment. On the other hand, 77.6% of non-Kuwaitis had surgery, 70.5% had chemotherapy; 65.9% had radiotherapy; and 67.5% had hormonal-based treatment [8].

The TRIPOLI study possibly provides a more valid description of current treatment strategies as it reports on more recent data collected between 2017 and 2019; it describes that out of the 387 TNBC cases from Oman, Kuwait, and Qatar, among other Arab countries, with non-metastatic disease who started treatment during that period, 217 patients (56.1%) had upfront surgery and 170 patients (43.9%) started with neoadjuvant chemotherapy [37]. 

### 6.2. Outcomes

Data on outcomes from the GCC were also scarce. Besides data from the GLOBOCAN database [3], only the UAE’s and Bahrain’s national registries [4,6] reported BC deaths (Table 1 and Table 2). Looking at original papers published from the five countries between 2011 and 2021, most analyzed datasets were of patients diagnosed before 2000 and up to the year 2012 [10,13,22,40,41,66,67,68,69], and one meta-analysis looking at survival rates in patients with BC from the Eastern Mediterranean region included data within the same timeframe [70]. Only a couple papers reported outcome data up to year 2015 [71,72] with the absence of any outcome data from the region thereafter.

## 7. Consensus and Recommendations from the GCC

During the past decade, we could find three expert opinion pieces involving a collaboration from the GCC: One was framed by the UAE Oncology Task Force in 2019 and outlined challenges and opportunities in BC care while proposing recommendations for policymakers and healthcare practitioners for the delivery of high-quality care to BC patients [73]. The other two [74,75] involved one expert from the UAE among other international experts from countries like Argentina, Brazil, Colombia, Egypt, Mexico, Moscow, and South Korea, Singapore, and Taiwan and were therefore not specific to the regional GCC context, and while one focused on treatment selection in HR+HER2− metastatic BC (mBC) [75], the second was more focused on the BRCA testing and management of HR+ HER2-mBC in the era of poly (ADP-ribose) polymerase inhibitors (PARPi) [74].

## 8. Conclusions and Implications

Our review of the BC literature primarily highlights the evolving role of national cancer registries in the GCC countries, given the inconsistent and incomplete reporting of BC data in the past and the very limited information they provide, which has been improving over last few years with better detailed reporting. In addition, there is an overall paucity of data, mostly around the epidemiology of BC in the GCC, treatment strategies, and management outcomes in BC, and the scarce available data are predominantly outdated and therefore not necessarily reflective of the current status of BC in the GCC, especially given that the approach for treating BC and, as a result, patient outcomes are continuously evolving with the emergence of new therapies. Adding to that, the adoption of treatment strategies is highly affected and driven by the availability of resources and financial assets, which in turn are variable across the six countries. The fact that the consistent reporting of data is lacking and that the reviewed data were captured from different time periods across the six countries makes intercountry comparisons almost impossible. 

It is explained that the lack of research in the Arab world is primarily attributed to the limited resources for research infrastructure in cancer institutions, modest governmental and nongovernmental funding, and suboptimal collaboration between various stakeholders [76]. Consequently, country- and regional-level strategies should be put in place to cope with the anticipated increase in BC cases. One aspect these strategies should focus on is the strengthening of health information systems, which will allow (1) a better assessment of BC burden in the region; (2) an improved understanding of patients and healthcare system demands in light of the anticipated increase in incidence; (3) a guided evaluation of adopted strategies in BC control and management based on specific indicators and metrics; and accordingly, (4) agile and evidence-based decision making so that decisions are tailored to the needs of patients in the region and that cancer management is optimized. The above is achieved through the proper allocation of budget and resources by the government toward the leveraging of national cancer registries so that not only do they actively collect accurate and reliable data around BC characteristics, management, and outcomes but that these data are made publicly available in a periodic manner for review and utilization by various stakeholders. In addition, efforts should be directed toward supporting research infrastructure to encourage investigator-led research around various topics in BC, with a focus on regional needs and priorities. Finally, it is imperative that industry and academia work closely together following a common agenda of research priorities in order to (1) ensure proper and sustainable funding for research activities and (2) avoid any unnecessary replication of research work and wasted efforts on topics of lesser importance.

## Figures and Tables

**Table 1:** Breast cancer epidemiology and clinicopathology data from the UAE, Qatar, Kuwait, Bahrain, Oman, and Saudi Arabia based on the GLOBOCAN database.

	The UAE	Qatar	Kuwait	Bahrain	Oman	Saudi Arabia
GLOBOCAN 2020 [3]						
Number of new cases	1030	218	791	244	558	3954
Percent of all cancers	21.4%	14.7%	20.6%	20.1%	15%	14.2%
Percent of all female cancers	38.8%	37.5%	39.5%	37.9%	37.7%	29%
Number of BC deaths	222	57	224	66	195	1095
Percent of all cancer deaths	11.7%	8.1%	13%	11.1%	9.6%	8.4%
5-year prevalence	3746 (122.66 per 100,000)	776 (108.39 per 100,000)	2805 (169.37 per 100,000)	868 (144.40 per 100,000)	1849 (106.49 per 100,000)	13,653 (92.99 per 100,000)
ASR incidence	58.5 per 100,000	42.7 per 100,000	50.3 per 100,000	44.2 per 100,000	38.5 per 100,000	28.8 per 100,000
ASR mortality	16.6 per 100,000	13.2 per 100,000	17.0 per 100,000	13.6 per 100,000	14.5 per 100,000	8.9 per 100,000

ASR, age-standardized rate; BC, breast cancer.

**Table 2:** Breast cancer epidemiology and clinicopathology data from the UAE, Qatar, Kuwait, Bahrain, Oman, and Saudi Arabia based on the reports from the countries’ respective national cancer registries.

	The UAE	Qatar	Kuwait	Bahrain	Oman	Saudi Arabia
National Cancer Registry						
Year	2019 [4]	2018 [5]	2017 [8]	2018 [6] (limited to Bahrainis)	2019 [7]	2015 [9]
Number of new cases	883 invasive, 95 in situ	367 87 (Qatari) 280 (Non-Qatari)	608	211	350 invasive, 12 in situ 362 (Omanis) 55 (non-Omanis)	2016 (Saudi) 718 (non-Saudi)
Percent of all cancers	20.2%	17.17%	21.8%	25%	16.7% (Omanis) 28% (non-Omanis)	16.7% (Saudi) 20.5% (non-Saudi)
Percent of all female cancers	36.1%		38.4%	40.7%	29.19% (Omanis) 68.75% (non-Omanis)	30.1% (Saudi) 41.4% (non-Saudi)
ASR incidence among females		87.07 per 100,000	61.0 per 100,000 (Kuwaiti) 41.3 per 100,000 (non-Kuwaiti)	66.0 per 100,000	35.4 per 100,000 (Omanis)	24.3 per 100,000 (Saudi) 29.8 per 100,000 (Non-Saudi)
ASR (all)	78.4 per 100,000		187.8 per 100,000			
Crude incidence rate		50.28 per 100,000		61.5 per 100,000	25.64 per 100,000 (Omanis)	20.3 per 100,000 (Saudi Females) 19.9 per 100,000 (non-Saudi Females)
Deaths		6 (Qatari)		68 (Females) 1 (Male)	43	
Percent of all cancer deaths	11.6%	22.92% (Qatari)		16.2%	8.9%	
Percent of all female cancer deaths				28.2%	20%	
Median age/Mean age			Kuwaitis: 55.9 Non-Kuwaitis: 49.9		48.5	Saudi: 50
Peak of incidence age		45–49 (*n* = 68 cases were diagnosed in this age group) (Qatari)		50–54 (*n* = 36 cases were diagnosed in this age group)		Saudi Female: 45–49 (*n* = 356 cases were diagnosed in this age group) Non-Saudi Female: 40–44 (*n* = 124 cases were diagnosed in this age group)
Histology		Infiltrating duct carcinoma: 80.45%, *n* = 288		Infiltrating ductal carcinoma: 88.0%	Infiltrating duct carcinoma: 90.3%	Saudi: Infiltrating duct carcinoma: 79.2%
Staging		Stage I: 13% Stage II: 46% Stage III: 33% Stage IV: 8%	Kuwaiti: Stage I: 17% Stage II: 29% Stage III: 20% Stage IV: 12% Unknown: 21% Tis: 1% Non-Kuwaiti: Stage I: 10% Stage II: 27% Stage III: 28% Stage IV: 16% Unknown: 16% Tis: 3%		Omani: Stage 0 (in situ): 4% Stage I: 27% Stage II: 34% Stage III: 17% Stage IV: 15% Unknown: 3% Non-Omani: Stage 0 (in situ): 2% Stage I: 29% Stage II: 32% Stage III: 3% Stage IV: 18% Unknown: 16%	Saudi: Distant: 15.0% Localized: 38.3% Regional: 38.6% Unknown: 8.2%
Treatment type		Top 3 treatment modalities: 1. Chemotherapy/Surgery: 23.20% 2. Surgery: 19.12% 3. Chemotherapy/Radiation Therapy/Surgery: 15.67%	Kuwaiti: Surgery: 82.3% Chemotherapy: 60.8% Radiotherapy: 65.8% Hormonal: 74.0% Non-Kuwaiti: Surgery: 77.6% Chemotherapy: 70.5% Radiotherapy: 65.9% Hormonal: 67.5%			
Survival			Kuwaiti: 1-year survival: 97.4% 3-year survival: 92.5% 5-year survival: 90.5%			

ASR, age-standardized rate; BC, breast cancer.
